# No effect of interspecific thermal tolerance on the stability of a blue mussel, *Mytilus* sp., hybrid zone

**DOI:** 10.1038/s41598-026-52835-7

**Published:** 2026-05-25

**Authors:** Jakob Thyrring, Morgane Touzot, Antoine Quennevat, Lis Bach, Jesper G. Sørensen

**Affiliations:** 1 Department of Ecoscience, Aarhus University, C. F. Møllers Allé 3 , 8000 Aarhus C, Denmark; 2 Department of Ecoscience, Aarhus University, Frederiksborgvej 399, 4000 Roskilde, Denmark; 3 Department of Biology, Aarhus University, Ole Worms Allé 1, 8000 Aarhus C, Denmark

**Keywords:** Arctic, Competition, Species distribution, Gene expression, Heat stress, Intertidal rocky shore, Ecology, Ecology, Ocean sciences

## Abstract

Arctic coastal ecosystems are undergoing rapid transformation as accelerated warming intensifies cryosphere melt and drives poleward range expansions of boreal species. In West Greenland, the blue mussels *Mytilus edulis* and *M. trossulus* form a distinct hybrid zone that may be destabilized in response to increasing temperatures and changing salinity regimes. To assess the impacts of warming and salinity stress on the hybrid zone stability, we conducted a mortality experiment. Mussels were exposed to three salinities (5, 15, or 25 PSU) for seven days before daily exposure to one of four air temperatures (5, 30, 33, or 36°C) for 1.5 hours over the course of six days, representative of conditions in the collection area. Survival was assessed daily, and we measured the expression of six stress-related genes (*hsp70, hsp90, sod, oat, p38*, and *p53*) in *M. edulis*, *M. trossulus*, and their hybrids after six simulated tidal regimes. Mortality increased significantly with increasing temperatures and decreasing salinity across all species, with >75% mortality at 36°C, but no interspecific differences in survival or gene expression were detected. Our findings suggest that atmospheric warming alone is unlikely to destabilize or shift the *M. edulis–M. trossulus* hybrid zone northward. Instead, other abiotic and biotic constraints, such as prolonged exposure to sub-zero temperatures, food availability, or reproductive limitations, may limit the poleward expansion of *M. edulis*. Our study provides the first experimental evidence of comparable thermal and salinity tolerance among co-occurring *Mytilus* congeners in the Arctic, highlighting the resilience of this hybrid zone under near-future climate scenarios.

**Supplementary Information:**

The online version contains supplementary material available at 10.1038/s41598-026-52835-7.

## Introduction

The Arctic atmosphere is warming faster than the ocean, and three to seven times faster than the global average^1^. The frequency and duration of Arctic heat waves have increased, and in West Greenland, the number of days with extreme sub-zero air temperatures decreased by more than 50% since the 1950s^2,3^. While negatively impacting Arctic species, warming is benefiting boreal species (e.g., through increased growth, abundance, and biodiversity) as their poleward ranges expand and new habitats emerge. The intrusion of boreal species has already had ecosystem-wide consequences, including altered community composition^4^, increased biomass and biodiversity^5^, and a reduced abundance of sea-ice-associated fauna (e.g., polar bear, walrus, and ice algae)^6^. Yet, aside from some local studies, knowledge of Arctic coastal biodiversity and species distribution remains limited, constraining the capacity to forecast future range shifts and ecosystem changes across regions^7,8^.

**Fig. 1:**
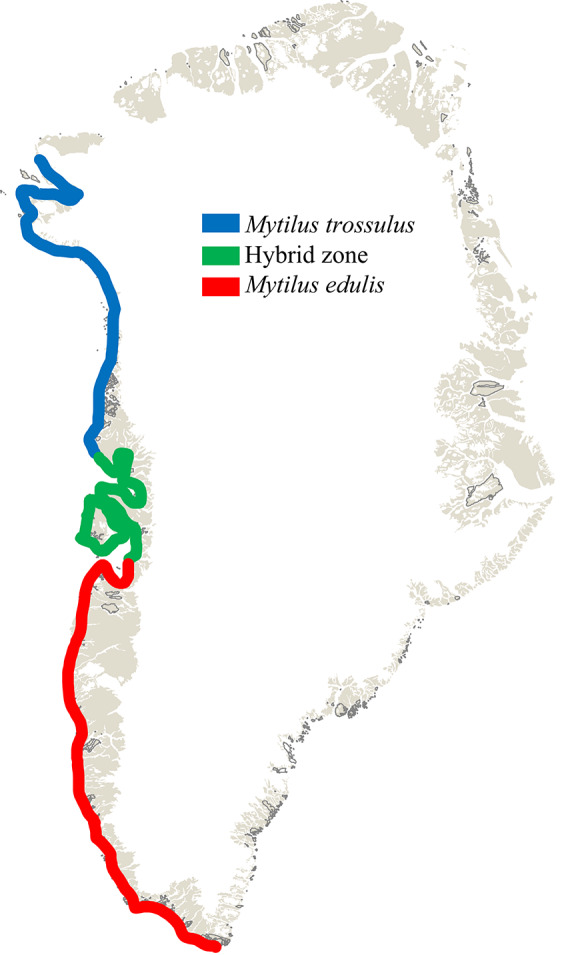
Regions in West Greenland dominated by *M. trossulus* (blue), *M. edulis* (red) and the intermediate *M. edulis-M. trossulus* hybrid zone (green). Black dot indicates the sampling site. Map generated using Adobe Illustrator (version CC 2014-18.0.0 - https://www.adobe.com/products/illustrator.html) and Microsoft PowerPoint (version 16.108.3 - https://www.microsoft.com/microsoft-365/powerpoint).

The *Mytilus* (blue mussel) species complex, comprising at least three species, *Mytilus edulis, M. trossulus,* and *M. galloprovincialis,* occurs intertidally throughout temperate and Arctic shorelines. Historically, only *M. edulis* was described in the Arctic; however, recent work has demonstrated the presence of multiple *Mytilus* spp. and their hybrids throughout the region^9,10^. In West Greenland, *M. edulis* and *M. trossulus* exhibit a distinct distributional pattern: *M. trossulus* dominates in the north, while *M. edulis* dominates in the south, separated by an intermediate hybrid zone where both species coexist and hybridize (Fig. 1)^11,12^. The performance of *Mytilus* species and hybrids varies geographically. For example, physiological differentiation maintains hybrid structure in a *M. edulis*-*M. galloprovincialis* zone of southwest England^13^, whereas in the *M. edulis*-*M. trossulus* hybrid zones of Newfoundland, Canada, and the Baltic Sea, hybrids generally do not outperform either parental species and often show reduced survivorship under environmental stress^9,14^. The persistence of the distributional pattern in West Greenland remains unclear, but recent genetic analyses show that this hybrid zone is bimodal and dominated by pure individuals, with hybrids found primarily as F1 crosses occurring in a narrow environmental niche, suggesting reduced hybrid fitness and limited persistence compared with parental species^9^. Thus, this newly identified hybrid zone offers a natural laboratory for investigating ecological responses among closely related species with distinct interspecific physiology and reproductive performance^14–16^.

The origin, structure, and stability of hybrid zones, as well as their responses to climate change, have long been a central topic in evolutionary ecology. Ecological theory predicts that hybrid zones should respond to factors that influence species’ distribution limits, such as changing climate and dispersal dynamics. Today, no pure *M. edulis* individuals have been detected north of the *M. edulis*–*M. trossulus* hybrid zone^10,12^, but the impacts of climate change may facilitate the expansion of *M. edulis* northward by larval transport from the southern populations with the north-flowing West Greenland Current, potentially displacing *M. trossulus.* Similar replacements have been documented in Europe, where *M. edulis* replaced *M. trossulus* (except in the Baltic Sea) ~8000 years ago^17^, and in California, USA, where the more heat-adapted *M. galloprovincialis* replaced *M. trossulus* during the 20th century^18^.

Like *M. galloprovincialis, M. edulis* generally exhibits a higher critical thermal limit than *M. trossulus* and their hybrids^19^, supporting the hypothesis that Arctic warming could shift the hybrid zone northward into the historical range of the cold-adapted *M. trossulus.* Both Greenland *M. edulis* and *M. trossulus* can tolerate water temperatures above 20°C^20,21^, well beyond projected future Arctic sea surface temperatures, indicating that ocean warming is unlikely to be a primary driver of their distribution. In contrast, intertidal species in Greenland periodically experience extreme air temperatures during low tide, where intertidal microhabitat temperatures can exceed 36°C^2^, which is above the physiological thermal tolerance of many intertidal species. Because heat stress in the Arctic intertidal zone is driven by aerial exposure rather than water temperature, air temperature represents the most ecologically relevant thermal stressor to investigate. Yet, little is known about the physiological consequences of aerial heat stress in intertidal species, particularly in combination with other stressors^22^. Previous work has shown that low-salinity conditions exacerbate the effects of air temperature stress in *M. edulis*^23,24^, and at lower latitudes, extreme air temperatures have caused mass mortality of intertidal species during low tides^25^, highlighting that atmospheric warming may be an overlooked determinant of abundance, biodiversity, and geographic limits in Arctic intertidal ecosystems.

Differences in physiology and habitat use may further mediate species responses among *Mytilus* spp. For instance, the production of chaperone proteins such as heat shock proteins (Hsps) regulates cellular stress responses^26,27^, and interspecific differences in the energetic costs of protein repair influence species fitness and distribution^28,29^. Furthermore, *M. trossulus* dominates freshwater-influenced inner fjord environments with salinities as low as 5 PSU^11,30^, suggesting adaptation to low-salinity conditions, as also observed elsewhere^31^. Therefore, by determining species-specific variation in mortality and the expression of heat shock proteins, this study investigates if air temperature stress is an important driver for Mytilus distribution in the Arctic. Specifically, we test the hypothesis that the cold-adapted *M. trossulus* and *M. edulis–M. trossulus* hybrids are more sensitive to combined thermal and salinity stress than *M. edulis*, potentially destabilizing the *M. edulis–M. trossulus* hybrid zone under future Arctic warming.

## Material and methods

### Sampling site and holding conditions

At the sampling site in Maarmorilik, West Greenland (71°06’41.8’’N 51°23’26.9’’W), summer seawater temperatures remain consistently low (<10°C), whereas air temperatures in sun-exposed intertidal microhabitats can reach 25–36°C during low tide. Thus, intertidal mussels naturally experience rapid transitions from cold-water immersion to brief periods of extreme aerial heating and return to cold water as the tide rises. To replicate these natural Arctic conditions and isolate the effects of aerial heat stress, all mussels were held in 5°C water between daily air-temperature treatments. Blue mussels (*Mytilus* spp.) were collected at an intertidal site known to contain both* M. edulis, M. trossulus,* and their hybrids on August 11, 2022. Mussels were immediately wrapped in moist towels, placed in cooling boxes containing frozen freezer blocks, and quickly, within two days, transported to holding facilities at Aarhus University, Denmark. Upon arrival at the holding facility, all mussels were kept in 5°C water in a cold room (5°C) for a seven-day adjustment period in aerated 10 L aquaria (25 PSU), and daily fed by adding 5 mL of microalgae solution (Shellfish Diet 1800, Reed Mariculture, USA) to each aquarium by pipetting directly into the water. The water was exchanged every second day to minimize the build-up of pseudofeces and excretion products.

### Experimental design

During summer, air and surface temperatures, including mussel beds, can exceed 36°C in Greenland’s intertidal zone^2^, while freshwater run-off from melting glaciers creates strong salinity gradients in fjord systems***,*** where the salinity can fall below 5 PSU^24,30,32^. To represent realistic conditions in Greenland’s intertidal zone, we designed a full-factorial experiment to determine the survival of *M. edulis, M. trossulus,* and their hybrids after exposure to four air temperatures (5, 30, 33, 36°C) combined with three different salinities (5, 15, 25 PSU), for a total of 12 treatments (Fig. S1). Despite some species-specific variation in shell morphology^33^, it is impractical to reliably distinguish *M. edulis*, *M. trossulus,* and their hybrids based on shell shape alone. Therefore, we initially allocated 15 individuals to the three groups (i.e., *M. edulis, M. trossulus,* or hybrids) based on their shell shape (following^33^), and after the experiment, gill tissue from each mussel was preserved individually in 95% ethanol for molecular species identification.

A total of 540 mussels (shell length 37 mm ± 6.1 SD) were used in the experiment (3 species x 3 salinities x 4 temperatures x 15 replicates). Mussels were exposed to three salinity levels (5, 15, and 25 PSU) for seven days at a constant water temperature of 5°C. Individuals were placed in 72 perforated 0.5 L plastic buckets, distributed across 12 aerated 10 L aquaria (four aquaria per salinity treatment). Each aquarium housed 45 mussels, 15 individuals per species, divided between two buckets for each species. Salinity conditions were reached by diluting the 25 PSU seawater with demineralized water. The salinity was checked daily with a handheld refractometer.

After seven days, mussels (n=15) from each salinity x species treatment were daily exposed to one of four air temperatures (5, 30, 33, or 36°C) for 1.5 h over the course of six days by placing them on trays inside a climate chamber preheated to the experimental temperature (Memmert GmbH, Germany). The 1.5-hour exposure time was selected to simulate realistic aerial exposure in the low intertidal zone where blue mussels in northern Greenland are most abundant^2^.

A temperature logger (iButtonlink Technology) was placed in each climate chamber to verify air temperature conditions. After each temperature treatment, all mussels were returned to holding aquaria water (5°C, salinity 5, 15, or 25 PSU depending on salinity treatment), and mortality was assessed by reaction to tactile stimuli. Dead mussels were removed from the experiment, and their shell size was measured using a digital calliper. At the end of the experiment, after the last air exposure, surviving mussels were allowed to recover in water (5°C and the treatment-specific salinity) for 1 h before gill tissue was dissected and snap-frozen using dry ice. Gill tissue was immediately stored at −80°C for later gene expression analysis. After tissue dissection, the shell length of all remaining mussels was measured.

### Species identification

Total DNA was extracted from 5–30 mg of gill tissues conserved in 95% ethanol using the E.Z.N.A Mollusc DNA kit (Omega Bio-tek, D3373-01, Norcross, GA, USA) according to the manufacturer’s protocol with slight adaptations. In brief, the tissue was homogenized in 350 µL ML1 Buffer (high-salt buffer containing cetyltrimethyl ammonium bromide) and 25 µL Proteinase K, incubated at 60°C for 30 min, and then 350 μL chloroform:isoamyl alcohol (24:1) was added. Polysaccharides and proteins were then removed by centrifugation of the samples at 10,000g for 2 min. One volume of BL Buffer and 10 µL of RNase A were added to the samples, which were then incubated at 70°C for 10 min to remove the RNA. DNA was then precipitated from the supernatant by the addition of one volume of 100% ethanol and further purified using HiBind® DNA Mini Column and the addition of 500 μL HBC Buffer. After two wash steps, DNA was pelleted by adding 700 µL of DNA Wash Buffer to the HiBind® DNA Mini Column and centrifugation at 10,000g for 1 min and air-dried by centrifugation at 10,000g for 2 min. Finally, DNA was dissolved in 50–100 µL Elution buffer preheated at 70°C. The resulting DNA was quantified by fluorescence using a Qubit 3.0 fluorometer (Qubit® 3.0 fluorometer, Invitrogen, Q33216) and the Quant Qubit dsDNA HS Assay Kits (Qubit® dsDNA HS Assay Kits Molecular Probes, Invitrogen, Q32854) and stored at −20°C until amplification.

For species identification, quantitative polymerase chain reaction (qPCR) was used to amplify a specific 168-bp segment for *M. trossulus*, a 180-bp segment for *M. edulis*, and these exact same two segments for the hybrids *M. edulis*–*M. trossulus* of the Glu gene (polyphenolic adhesive protein), which is a reliable method to differentiate between *Mytilus* spp. and hybrids^34–38^. The 20-µL qPCR reaction contained 1 µL DNA template, 1 µL of each 10 µM forward (M15 - CCAGTATACAAACCTGTGAAGA) and reverse (M16 - TGTTGTCTTAATAGGTTTGTAAGA) primer, 10 µL of SsoFast EvaGreen supermix (SsoFast™ EvaGreen^®^ Supermix, BIO-RAD, 172–5201), 1 µL of BSA, and 6 µL of nuclease-free water. Samples were run on a real-time thermal cycler Stratagene MxPro – MX3005P qPCR system (Stratagene Mx3005P, Agilent) with the following program: initial denaturation and enzyme activation for 3 min at 98°C, followed by 40 cycles of 10 s at 95°C, 20 s at 56°C and 20 s at 72°C, then a final extension step of 7 min at 72°C. Finally, a high-resolution melting curve was obtained by gradually increasing the temperature from 72°C to 95°C (0.1°C per 10 s). Because the size difference between the PCR products of *M. edulis* and *M. trossulus* was small, we decided to distinguish the pure species and hybrids based on melting peaks. A unique peak around 79.5°C and 81°C was observed for *M. trossulus* and *M. edulis,* respectively, while two peaks were observed for the hybrids*.*

### Gene expression analysis

Expression of two heat shock protein genes, *hsp70* and *hsp90*, as well as four antioxidant and detoxifying enzyme genes, superoxide dismutase* (sod)*, ornithine aminotransferase (*oat*), p38 mitogen-activated protein kinase *(p38),* and tumor suppressor protein p53 *(p53)*, was analysed (Table 1). The RNA was isolated from gill tissue from *M. edulis* (n = 29), *M. trossulus* (n = 42), and hybrids (n = 10) across six treatment combinations: 5 °C–5 PSU, 5 °C–15 PSU, 30 °C–15 PSU, 30 °C–25 PSU, 33 °C–15 PSU, and 33 °C–25 PSU. The unbalanced sample sizes resulted from post-experimental species identification. Total RNA extraction was performed using the E.Z.N.A Total RNA kit I (Omega Bio-tek, Norcross, GA, USA) according to the manufacturer’s instructions. RNA concentration was determined using Invitrogen Qubit™ fluorometer. cDNA synthesis was performed on 1000 ng of the total RNA using an AffinityScript cDNA Synthesis Kit (Agilent Technologies) according to the manufacturer’s instructions, and the derived cDNA eluate was diluted to a concentration of 4 ng/µL. Primer sequences of the six target genes are presented in Table 1, and gene expression of these six genes in mussels was quantified using qPCR. The qPCR was performed on a Stratagene MxPro – MX3005P qPCR system (AH Diagnostics, Denmark) using a standard two-step thermal profile followed by a dissociation curve. Brilliant II SYBR® Green qPCR Master Mix (Agilent Technologies) and ROX as reference dye were used, with 10 µL mastermix and 5 µL template per reaction. For every biological replicate, technical duplicates were performed. The amplification protocol consisted of 95°C for 10 min, then 40 cycles of 95°C for 30 s and 60°C for 60 s, followed by determining a melting curve. Amplification plots and dissociation curves were visually analyzed to remove failed amplifications from the dataset.

**Table 1:** Genes tested in the gene expression analysis, their expected target response, and primer sequences.

**Gene**	**Expected target response**	**Primer sequence**	**Reference**
*hsp70*	Temperature/salinity	Forward primer: GGTGGTGAAGACTTTGACAACAG Reverse primer: CTAGTTTGGCATCACGTAGAGC	(GenBank: AF172607.1)^41^
*hsp90*	Temperature	Forward primer: CCAGCTGCTGATTCCCAGAT Reverse primer: TGGTGTAGGTTTCTACTCCGC	(GenBank: JZ970419.1, AJ586906.3)
*Oat*	Salinity	Forward primer: ATCTCATCATCTACAGACCCTTCAA Reverse primer: ACATTGGGATCTTGGAATTCTCTCT	(GenBank: OK546225, OK546229, OK546228)^42^
*Sod*	Oxidative stress	Forward primer: AGCTATCCCTGACTGGTCCC Reverse primer: TTGCTTAGCTCATGGCCACC	(GenBank: AJ581746.1, FM177867.1)
*p38*	Salinity	Forward primer: CATTGGAGCGAATCCTCTTGC Reverse primer: GCATCTTCTGCAGTGACACG	^24^
*p53*	Cell Cycle/apoptosis	Forward primer: TGTCATCCATGGTTCCCCAG Reverse primer: CTCCCTGGCCAGTATCATCG	(GenBank: AY579472.1, AY611471.1)

The amplification data were used to estimate the relative cDNA content before amplification (R_0_, corresponding to the original relative RNA content) using Data Analysis for Real-Time PCR (DART-PCR)^39^. Generally, gene expression data are normalized using one or several reference genes, which assume that the reference genes are unaffected by the treatments and are measured accurately and consistently. These two assumptions cannot always be met. Thus, we normalized the R_0_ data using the NORMA-Gene method^40^, which provides target-data-driven normalization of R_0_ values among biological replicates and across genes, without relying on invariable reference genes. The fold change (FC) values are presented relative to the *M. trossulus* 5 °C-5 PSU treatment for each gene.

### Statistical analyses

We investigated the effects of salinity and air temperature on survival using generalized linear models (GLMs) with a binomial distribution and a logit link function. Final models were validated by plotting residuals against fitted values and against each covariate in the model. Validation models indicated no violation of model assumptions. For each species, ANOVA was used to test for differences in gene expression among temperature-salinity treatments. Visual inspection of Q-Q plots and residuals was used to validate ANOVA models. To evaluate the statistical power of our mortality experiment, we conducted a simulation-based sensitivity analysis. Using the observed mortality probabilities for each Species × Temperature × Salinity combination, we simulated datasets in which *M. trossulus* exhibited increased mortality relative to *M. edulis* and hybrids. We applied additive logit-scale effect sizes corresponding to 1.5×, 2×, and 3× higher odds of mortality and generated 1,000 simulated datasets per scenario. For each simulation, we fitted binomial GLMs with and without the species term and quantified power as the proportion of likelihood-ratio tests detecting a significant species effect. This approach allowed us to estimate the effect sizes that could be reliably detected given the sample sizes available. The level of significance in all tests was p < 0.05. All analyses were conducted using R^43^.

## Results

### Species identification

The method of DNA amplification and melting curve analysis of mussel gill tissue successfully distinguished between the two pure species and their hybrids, with the majority of individuals being *M. trossulus* (67%), followed by *M. edulis* (19%) and hybrids (14%). The proportion of *M. edulis, M. trossulus,* and hybrids was not equally distributed across treatments (Supplementary Table S1), and for the survival experiment, we excluded species-specific treatments with less than five individuals (*M. edulis* 5 °C-15 PSU, 5 °C-25 PSU, 36 °C-15 PSU, and hybrids 30 °C-15 PSU, 30 °C-25 PSU, 33 °C-5 PSU, 33 °C-15 PSU) from the final analyses.

### Survival

**Fig. 2:**
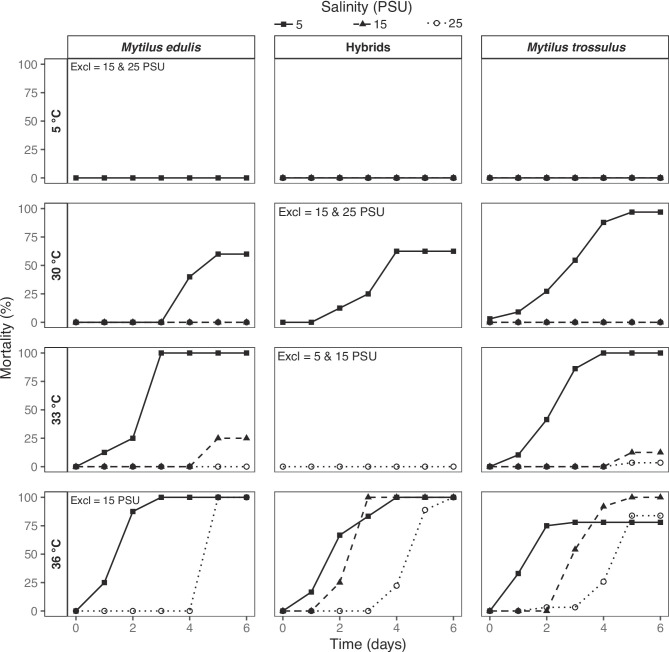
Mortality (%) in *M. edulis, M. trossulus,* and their hybrids as a function of time (days). Mussels were acclimated to three salinities (5, 15, and 25 PSU) for seven days and subsequently exposed to one of four air temperature treatments (5, 30, 33, and 36°C) for 1.5 h daily over six consecutive days. “Excl” refers to salinity treatments that have been excluded from the statistical and graphical data set. For treatment sample sizes, see Supplementary Table S1.

Increasing temperatures (F_3,25_ = 331.6, *p* < 0.001) and decreasing salinities (F_2,23_ = 267.8, *p* < 0.001) significantly reduced the survival of all three *Mytilus* species after six days of air exposure. No mortality was observed at 5°C across any salinity treatment. At 30°C, mortality occurred only at 5 PSU, with *M. trossulus* exhibiting a non-significant trend of higher mortality than *M. edulis* and hybrids (Fig. 2). After six days of exposure to 33°C, 25% of *M. edulis* acclimated to 15 PSU perished compared to 12.5% of *M. trossulus* (Fig. 2). At 36°C, the mortality rate was >75% across all salinity treatments, and over time mortality increased in a similar manner in the three species. Individuals from the 36 °C-5 PSU treatment were most susceptible to repeated heat stress, displaying a marked increase in mortality after only two emersion cycles. In contrast, mussels from the 36 °C-25 PSU treatment displayed low mortality (0–25%) until after four emersion cycles (Fig. 2). It should be noted that the simulation-based sensitivity analysis revealed low statistical power to detect modest species-specific differences in mortality. When *M. trossulus* was assigned 1.5× higher odds of mortality than *M. edulis* and hybrids, the power was 0.11. A twofold difference yielded moderate power (0.49), while a threefold difference resulted in high power (0.99).

### Gene expression

**Fig. 3:**
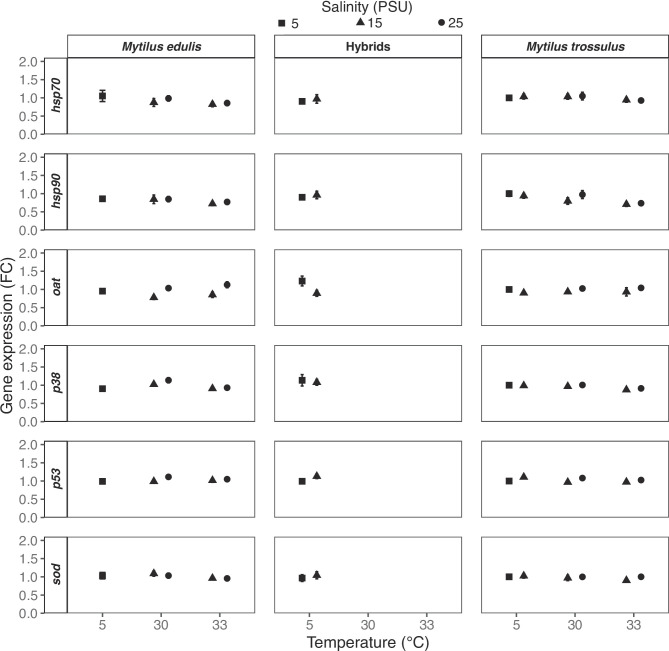
Average change in gene expression (fold change, FC) of *hsp70, hsp90, oat, p38, p53,* and *sod* in *M. edulis, M. trossulus,* and their hybrids. Mussels were acclimated to three salinities (5, 15, and 25 PSU) for seven days and subsequently exposed to one of four air temperature treatments (5, 30, 33, and 36°C) for 1.5 h daily over six consecutive days. FC values are presented relative to the *M. trossulus* 5 °C-5 PSU treatment for each gene. Dots represent mean values, and error bars represent standard error. Treatments with less than three samples were excluded. For specific treatment sample sizes, see Supplementary Table S2.

For gene analyses, all treatments with fewer than three samples were excluded. No species-specific differences were detected in the expression of the six genes of interest (*hsp70, hsp90, oat, p38, p53,* and *sod*) studied (Fig. 3).

## Discussion

The effect of temperature on physiological processes plays a fundamental role in determining the distribution and abundance of species. In the Arctic, blue mussels (*Mytilus* sp*.*) have been expanding northwards in response to warming^44^, and their abundance is predicted to increase in a warmer future^2^. In West Greenland, the distribution of *M. edulis* and *M. trossulus* is separated by an intermediate hybrid zone, and to test the stability of this hybrid zone, we investigated whether exposure to realistic air temperatures in combination with salinity stress induced interspecific differences in survival and expression of stress-related proteins among *M. edulis, M. trossulus*, and their hybrids.

Intertidal *Mytilus* sp. are highly resilient to temperature stress^45,46^, but repeated exposure to high air temperatures has been shown to decrease thermal tolerance in *M. edulis*^24^. Our results demonstrate that repeated exposure to high air temperatures also suppressed the thermal tolerance of *M. trossulus* and *M. edulis–M. trossulus* hybrids, as no interspecific differences in survival or the expression of six stress-related genes were observed. In all three species, the upper thermal limit was reduced by salinity, both in terms of air temperature and number of exposures required to induce mortality. Following exposure to low salinity (5 PSU) and 36°C, marked mortality was observed after two days, compared to four days in mussels acclimated in 25 PSU seawater. These results support previous work on the interactive effect of salinity and temperature on other marine ectotherms^47–50^. Our experimental design applied an acute temperature exposure to simulate rapid transitions from cold seawater to warm aerial conditions during low tide. In Greenland’s intertidal zone, tidal retreat can occur quickly, and mussels may experience sudden exposure to sun-heated substrates. However, heating rates in natural environments vary with factors such as solar radiation, wind exposure, substrate type, humidity, and microhabitat orientation. While our approach isolates the effects of acute thermal stress, future studies incorporating different heating regimes (e.g., gradual ramping vs. acute exposure) would improve our understanding of how warming influences thermal tolerance under more variable and realistic field conditions.

The post-experiment genetic identification revealed a highly uneven distribution of congeners across treatments. Consequently, the sample size for the mortality experiment was reduced to five, which is lower than other studies on thermal tolerance^51–54^, restricting the robustness of the results. This was also shown by the sensitivity analysis that indicated low power to detect species differences in mortality. Thus, the absence of species-specific effects should be interpreted cautiously, but the results still provide a valuable first insight into how climate change may affect the stability of this Arctic hybrid zone, particularly given the logistical challenges of studying morphologically similar species in remote environments. Considering that individuals from each of the three species survive repeated exposure to 36°C, and that *Mytilus* spp. in Greenland predominately inhabit microhabitats between boulders, in crevices, and underneath macroalgae fronts, which reduces extreme air temperatures^55–57^, our results indicate that atmospheric warming is of limited importance for the stability of the West Greenland *M. edulis–M. trossulus* hybrid zone. This conclusion was supported by gene expression results, which showed no species-specific differences, and the variation in fold change of stress-related genes was generally low compared to other studies on specific *Mytilus *sp.^24,58,59^. However, the observed fold changes in gene expression may be partially influenced by our choice of conserved gene sequences, which were designed to work across multiple *Mytilus* spp. Primers targeting consensus regions may reduce amplification efficiency in certain species or alleles, particularly in hybrids, and may fail to capture highly variable or species-specific transcript variants. As a result, the measured fold changes could underestimate the actual differences in expression. Alternatively, any stress-induced upregulation of RNA may have already returned to pre-stress levels before the gill tissue was collected (1h after the last aerial exposure), as RNA downregulation can occur within minutes to hours after stress^60^. While elevated *hsps* expression levels have previously been observed 1 h after exposure to stressful conditions in *Mytilus* and other aquatic species^59,61^, future studies should collect samples at regular time intervals during recovery to better capture these dynamics in various *Mytilus* spp. and hybrids.

*Mytilus* hybrid zones are well-known from lower latitudes^62–66^. However, as Arctic coastal species face a complex mosaic of climate drivers that can exacerbate the impacts of climate change^67^, the West Greenland *M. edulis–M. trossulus* hybrid zone is valuable for understanding the stability of marine hybrid zones in a changing climate. Processes other than high air temperatures may shape the current location of the hybrid zone. For example, the northern limit of *M. edulis* could be controlled by exposure to sub-zero air or water temperatures, combined with extended periods of darkness and low phytoplankton availability. For instance, water temperatures below 7°C suppress gametogenesis in *M. edulis*^68^*,* while the gametogenesis of *M. trossulus* in North Greenland is comparable to that of *M. edulis* populations from the subarctic^68,69^. Furthermore, several High Arctic bivalve species (including *M. trossulus*) feed on alternative food sources, including detritus, diatoms, and bacteria, providing energy throughout the polar night^70,71^. Because thermal tolerance in intertidal *Mytilus* spp. is influenced by food availability, with limited rations reducing survival and increasing heat-stress sensitivity^22,72^, interspecific differences in nutritional requirements may shape their responses to warming. In addition, early life-history stages may be more sensitive to stress compared to adults. Interestingly, *M. edulis* larvae show higher thermal tolerance than *M. trossulus* larvae^73^, and hybrid larvae often grow more slowly than pure species^74^, but it has also been shown that intraspecific variation in larval performance can vary as much among species^75^. Such differences in larval tolerance and developmental performance may ultimately influence dispersal, recruitment, and the future stability of the hybrid zone. Yet, interspecific physiological differences at different life stages and the use of alternative food sources by *M. edulis* and hybrids in the hybrid zone remain unexplored, and further research is needed to assess their physiological capacity to expand northward in Greenland.

The morphological similarity of *Mytilus* spp. is a challenge in physiological experiments. Numerous studies have tried to use morphological traits for *Mytilus* spp. identification^33,76–78^, but genetic identification using mtDNA markers, microsatellites, or nucleotide polymorphisms remains the only fully reliable method for genotyping *Mytilus* spp. This was evident in our study where *M. trossulus* comprised the majority of individuals (67%), while *M. edulis* and hybrids constituted 19% and 14%, respectively. Non-lethal gill sampling has been demonstrated in scallops^79^, and the use of pre-experimental non-lethal tissue sampling for genetic identification in *Mytilus* spp., should be explored in future studies to improve experimental design and reduce uneven sample distributions. Still, this study is the first to investigate interspecific physiological differences among *Mytilus* spp. from a single site in Greenland, and the overall data provide key insight into the stability and driving factors of the West Greenland *M. edulis–M. trossulus* hybrid zone. The low abundance of *M. edulis* could indicate a lower performance relative to *M. trossulus* (discussed above), or that *M. edulis* has recently expanded northwards in response to the increased frequency of Arctic heat waves^80^. Heat waves can indeed enhance northward range expansions of species into new regions^81^. For example, on Svalbard, *Mytilus* sp. reappeared in 2004 after being absent for around 1000 years^44^, and several fish species, including cod and herring, expanded their distribution along the west coast of Greenland during a period of elevated water temperatures in the 1930s^82^. A powerful tool to investigate range shifts and the historical distribution of species is the analysis of shells or bones from museum collections. For bivalves, recent advances in DNA technologies have enabled the extraction of DNA from ancient shells^83–85^, allowing for the reconstruction of past distributions and providing insights into the timing and pace of *M. edulis* distribution in Arctic environments.

## Supplementary Information

Supplementary Information.

## Data Availability

The datasets and R code used during the current study are available on Zenodo.org at https://zenodo.org/records/18171036
