# Supplementary material for: No effect of interspecific thermal tolerance on the stability of a blue mussel, Mytilus sp., hybrid zone
Source: Sci Rep. 2026 May 25;16:23828. doi: 10.1038/s41598-026-52835-7 (PMC13433996; doi:10.1038/s41598-026-52835-7)
Supplement: Supplementary file 1 [file 41598_2026_52835_MOESM1_ESM.docx]

Supplementary material

**No effect of interspecific thermal tolerance on the stability of a blue mussel, *Mytilus* sp., hybrid zone**

Jakob Thyrring^1,*^, Morgane Touzot^1^, Antoine Quennevat^1^, Lis Bach^2^, Jesper G. Sørensen^3^

^1^Aarhus University, Department of Ecoscience, C. F. Møllers Allé 3, 8000 Aarhus C, Denmark

^2^Aarhus University, Department of Ecoscience, Frederiksborgvej 399, 4000 Roskilde, Denmark

^3^Aarhus University, Department of Biology, Ole Worms Allé 1, 8000 Aarhus C, Denmark

**Corresponding author:** [thyrring@ecos.au.dk](mailto:thyrring@ecos.au.dk)

**Content:**

**Table S1:** Distribution of species across treatments …………………………………………… p. 2

**Table S2:** Sample size for gene expression analysis	…………………………………………… p. 2

**Figure S1:** Schematic illustration of the experimental design ……………………………..p. 3

**Table S1:** The final distribution of *M. edulis*, *M. trossulus*, and *M. edulis–M. trossulus* hybrids across the salinity–temperature treatments following species identification.

|  | *Mytilus edulis* | | | Hybrids | | | *Mytilus trossulu*s | | |
| --- | --- | --- | --- | --- | --- | --- | --- | --- | --- |
| Temperature/Salinity (PSU) | **5** | **15** | **25** | **5** | **15** | **25** | **5** | **15** | **25** |
| **5 °C** | 5 | 2 | 3 | 12 | 10 | 10 | 30 | 32 | 29 |
| **30 °C** | 5 | 17 | 13 | 8 | 3 | 1 | 32 | 27 | 32 |
| **33 °C** | 16 | 16 | 13 | 1 | 2 | 5 | 29 | 24 | 29 |
| **36 °C** | 8 | 3 | 5 | 6 | 8 | 9 | 33 | 31 | 31 |

**Table S2:** Samples sizes for the expression of six genes (*hsp70, hsp90, oat, p38, p53,* and *sod*) analysed in *M. edulis, M. trossulus* and *M. edulis–M. trossulus* hybrids across six temperature-salinity treatments. Treatments with less than three samples were excluded (n < 3)

| *Mytilus edulis* | | | | | | |
| --- | --- | --- | --- | --- | --- | --- |
| Treatments | *hsp70* | *hsp90* | *oat* | *p38* | *p53* | *sod* |
| 5 °C – 5 PSU | 3 | 3 | 3 | 3 | 3 | 3 |
| 5 °C – 15 PSU | 0 | 0 | 0 | 0 | 0 | 0 |
| 30 °C – 15 PSU | 5 | 5 | 5 | 5 | 5 | 5 |
| 30 °C – 25 PSU | 6 | 6 | 6 | 6 | 6 | 6 |
| 33 °C – 15 PSU | 9 | 9 | 9 | 9 | 9 | 9 |
| 33 °C – 25 PSU | 6 | 6 | 6 | 6 | 6 | 6 |
| Hybrids | | | | | |  |
| Treatments | *hsp70* | *hsp90* | *oat* | *p38* | *p53* | *sod* |
| 5 °C – 5 PSU | 3 | 3 | 3 | 3 | 3 | 3 |
| 5 °C – 15 PSU | 5 | 5 | 5 | 5 | 5 | 5 |
| 30 °C – 15 PSU | |  |  |  |  |  |
| 30 °C – 25 PSU | |  |  |  |  |  |
| 33 °C – 15 PSU | 1 | 1 | 1 | 1 | 1 | 1 |
| 33 °C – 25 PSU | 2 | 2 | 2 | 2 | 2 | 2 |
| *Mytilus trossulus* | | | | | |  |
| Treatments | *hsp70* | *hsp90* | *oat* | *p38* | *p53* | *sod* |
| 5 °C – 5 PSU | 8 | 8 | 8 | 8 | 8 | 8 |
| 5 °C – 15 PSU | 9 | 9 | 9 | 9 | 9 | 9 |
| 30 °C – 15 PSU | 7 | 7 | 7 | 7 | 7 | 7 |
| 30 °C – 25 PSU | 7 | 7 | 7 | 7 | 7 | 7 |
| 33 °C – 15 PSU | 5 | 5 | 5 | 5 | 5 | 5 |
| 33 °C – 25 PSU | 6 | 6 | 6 | 6 | 6 | 6 |

Figure S1: Schematic illustration of the experimental set-up. Step1: All mussels were kept for seven days at a salinity of either 5, 15 or 25 for seven days at 5 °C. After salinity acclimation, mussels were daily exposed to one of four air temperatures (Step 2) for six days. After each air exposure, the animals were transferred back in water at the treatment salinity and 5 °C.
